# Longitudinal Associations Between Biomarkers and Frailty in Older Adults: Protocol for a Scoping Review

**DOI:** 10.2196/83312

**Published:** 2026-03-24

**Authors:** Jo Woon Seok, Kyusol Shim, Yihan Yang, Soomin Hong

**Affiliations:** 1 Department of Basic Nursing Science College of Nursing Korea University Seongbuk-gu, Seoul Republic of Korea; 2 College of Nursing Ewha Womans University Seodaemun-gu, Seoul Republic of Korea; 3 Red Cross College of Nursing Chung-Ang University Dongjak-gu, Seoul Republic of Korea

**Keywords:** frailty, biomarkers, aged, longitudinal studies, systematic review

## Abstract

**Background:**

Frailty is a progressive, dynamic clinical syndrome characterized by reduced physiological reserves and increased vulnerability to stressors, leading to adverse outcomes in older adults. Despite its clinical significance, routine detection of frailty remains challenging owing to subtle presentations and time constraints during assessments. Longitudinal studies are essential for capturing its biological trajectory and identifying early biomarkers.

**Objective:**

This scoping review aims to systematically identify and map biomarkers—including inflammatory, hormonal, metabolic, genetic, and imaging-based markers—investigated for their longitudinal associations with frailty in older adults. Studies involving older adults with comorbid conditions (eg, cancer, infectious diseases, cardiovascular diseases) will also be included when frailty is assessed as an outcome.

**Methods:**

We will conduct a comprehensive literature search in 6 databases (PubMed, CINAHL, Cochrane Library, Scopus, Web of Science, and Embase). We will include longitudinal studies that examine the association between objectively measured biomarkers and frailty, as assessed with validated tools, in older adults. Studies published in English, Korean, or Chinese will be considered, with no restriction on publication date.

**Results:**

We identified 3315 records after duplicate removal in an exploratory search conducted in July 2024, indicating a substantial body of literature for review. We plan to complete this review by December 2026.

**Conclusions:**

This review will identify and describe longitudinal biomarkers that precede frailty in older adults, highlighting their potential for personalized clinical application. Anticipated limitations include reliance on observational study designs, methodological heterogeneity, and limited causal inference.

**International Registered Report Identifier (IRRID):**

PRR1-10.2196/83312

## Introduction

### Background

The rapid growth of the global older adult population has made frailty an emerging challenge in geriatric medicine and public health [1]. Frailty is a clinical syndrome, distinct from disability or comorbidity, characterized by reduced physiological reserves. This reduction increases vulnerability to stressors and results in functional decline in daily activities [2,3].

Frailty in community-dwelling older adults has been associated with adverse clinical outcomes, including falls, disability, hospitalization, and death [4,5]. It also negatively affects the health of individuals with cardiovascular disease, infectious diseases (eg, COVID-19, pneumonia), or cancer because it strongly predicts treatment intolerance [6], more frequent complications [7], and increased mortality [8]. These outcomes emphasize the clinical importance of timely frailty identification in older adults and suggest that biomarkers may improve early detection strategies [9,10].

Although frailty is clearly associated with adverse outcomes, routine detection remains challenging because of subtle or nonspecific presentations and time constraints during assessments, particularly in older adult care [8,11-13]. The multisystem and dynamic nature of frailty reduces the effectiveness of single-indicator approaches. This highlights the need for objective, scalable biomarkers that enable rapid and reproducible identification. In recent years, the unmet need has led to increased interest in biological markers that may offer a more accurate and less subjective approach to predicting, diagnosing, and monitoring frailty in older adults [14-16]. Several systematic reviews and meta-analyses have shown that the levels of cytokines, including interleukin-6, tumor necrosis factor-α, and C-reactive protein, are consistently elevated in frail older adults [10,17]. Beyond cytokines and inflammation, interest has increased in hormonal, metabolic, and molecular markers, as well as multimarker panels. Omics-based approaches (proteomics and transcriptomics) have been proposed to more accurately capture the heterogeneous and dynamic trajectories of frailty than any single biomarker. European consortia such as the FRAILOMIC initiative are conducting comprehensive profiling of genomic, proteomic, and clinical biomarkers to improve the prediction of frailty onset and progression, as well as the potential therapeutic response [18,19].

Despite growing interest in frailty biomarkers, most previous studies have been cross-sectional, primarily comparing biomarker levels between frail and robust individuals at a single point in time [17,20]. These explorations have highlighted inflammatory and metabolic dysregulation; however, because they rely on static measurements, they cannot capture biomarker dynamics or clarify whether these changes precede or result from frailty progression. This limitation reinforces the need for longitudinal evidence to delineate causal pathways and identify biomarkers that predict frailty trajectories.

Clinical inconsistency in frailty definitions, biomarker assays, and analytical techniques has further increased heterogeneity across studies [14,19]. For example, frailty has been operationalized using different instruments such as the Fried phenotype (which focuses on physical manifestations) and the Frailty Index (which aggregates deficits across multiple domains), leading to discordant classifications of the same individuals [14]. Similarly, inflammatory biomarkers such as interleukin-6 and C-reactive protein have been measured using assays with varying sensitivities (eg, standard enzyme-linked immunosorbent assay vs high-sensitivity assays) and inconsistent cutoff values, complicating cross-study comparisons [19]. These discrepancies have impeded the comparability and replication of the findings. The performance of biomarkers has been erratic because sensitivity and specificity have been affected by complicating factors, including comorbidity, drug use, and population variation. Large-scale longitudinal studies remain limited, which restricts our understanding of dynamic biomarker trajectories and their predictive value [21,22]. Consequently, no single biomarker panel has demonstrated sufficient robustness for routine clinical use in identifying frailty in older adults [23]. These gaps indicate the need for a systematic synthesis of longitudinal evidence to clarify consistencies, identify weaknesses, and guide future research.

### Objectives

This scoping review will systematically map longitudinal evidence on biomarkers associated with frailty in older adults. Existing evidence has identified the relevance of inflammatory and molecular markers; however, most studies have focused on single biological pathways or relied on cross-sectional associations. In contrast, we aim to describe findings across a broader range of biomarker types to reflect the biological complexity of frailty. Rather than following a hypothesis-driven approach, we will present a structured framework for interpreting and classifying diverse biomarkers (eg, inflammatory markers, hormonal mediators, genetic signatures, imaging-based measures) evaluated across diverse longitudinal study designs (eg, prospective cohort studies, follow-up assessment, and nested case-control analyses) and various demographic groups (eg, sex/gender, age strata, and clinical subpopulations such as heart failure or chronic kidney disease cohorts). This synthesis aims to identify clinically promising biomarkers, describe consistent trends, reveal key knowledge gaps to inform future research, and provide evidence-based suggestions for developing personalized frailty care strategies.

## Methods

### Study Design

We will conduct a scoping review to identify and review biomarkers with longitudinal associations with frailty, following the Joanna Briggs Institute (JBI) Manual for Evidence Synthesis guidelines [24]. We will report the review in accordance with the PRISMA-ScR (Preferred Reporting Items for Systematic Reviews and Meta-Analyses Extension for Scoping Reviews) guideline to ensure transparency and methodological rigor [25]. The review questions are as follows:

1. What types of biomarkers have been reported to be longitudinally associated with frailty in older adults?
2. What are the follow-up durations used in studies examining the association between biomarkers and frailty?
3. How do variations in longitudinal study design, frailty definitions and assessment tools, and key demographic factors (eg, age and sex) differ across studies?
4. What statistical methods have been employed to analyze the relationship between biomarkers and frailty over time?

### Eligibility Criteria

The review will include studies that meet the following criteria, structured according to population, concept, and context formats.

#### Population

Individuals aged 60 years or older with frailty were evaluated using defined tools, including the Frailty Index, Fried Frailty Phenotype, or laboratory-based frailty indices. Frailty was measured as a dependent variable rather than as a risk factor for other health outcomes such as mortality, hospitalization, and disability.

#### Concept

Biomarkers are defined as objective measures that represent cellular and organismal phenomena in the human body at a specific time [26]. However, the review will include studies that measured biomarkers directly reflecting the physiological mechanisms of frailty or its risk factors based on the pathogenesis of frailty [27,28]. Biomarkers of interest include hormones, inflammatory proteins, gene expression, and imaging markers. Studies that assessed biomarkers using methods not based on biological fluids (eg, body composition data from InBody or bioelectrical impedance analysis) were excluded to maintain consistency in biomarker definition and measurement approaches; however, imaging biomarkers such as functional magnetic resonance imaging (fMRI), resting-state fMRI, or diffusion tensor imaging will be included when the data capture neural function related to brain activity. Acceptable biological fluids include blood (eg, serum, plasma, whole blood for DNA/RNA analysis), urine, and other accessible fluids such as saliva.

#### Context

A study will be classified as longitudinal if it includes at least one follow-up frailty assessment in addition to the baseline evaluation and if biomarker measurements were obtained at baseline with frailty assessed at one or more later time points. This temporal ordering ensured prospective assessment of frailty incidence or progression.

### Search Methods

#### Sources of Evidence

This scoping review will include only peer-reviewed original research articles. We will also exclude reviews, qualitative studies, case studies, posters, abstracts, editorials, books, and commentaries because they lack original data or sufficient methodological details required to evaluate longitudinal associations between biomarkers and frailty.

#### Search Strategy

We will search 8 databases (PubMed, CINAHL, Cochrane Library, Scopus, Web of Science, Embase, CNKI, and RISS) by using key concept words such as “frailty,” “biomarkers,” and “longitudinal studies.” The search will have no date restrictions and will include literature published in Korean, English, or Chinese. We will not impose limitations on the study setting or geographic location.

The search strategy was initially developed using core controlled vocabularies from major databases, including Medical Subject Headings (MeSH) in PubMed, Subject Headings in CINAHL, and Emtree terms in Embase. Based on these controlled terms, we supplemented additional keywords relating to specific biomarkers and frailty phenotypes to construct the final search trails.

Two reviewers (KS and YY) independently drafted and refined the initial list of search terms, resolving any discrepancies through discussion. A third reviewer (JWS) subsequently evaluated the proposed terms and adjudicated remaining disagreements regarding the inclusion or exclusion of specific keywords. Following this adjudication process, a fourth reviewer (SH) performed a final review to ensure the completeness, accuracy, and coherence of the search terms before finalization. The details of the preliminary search trails for each database are presented in the Multimedia Appendix 1.

### Study Selection

We will import all retrieved citations into Covidence (Veritas Health Innovation Ltd, Melbourne, Australia) for reference management, with duplicates automatically removed. Two independent reviewers (KS and YY) will screen titles and abstracts to assess eligibility according to predefined inclusion and exclusion criteria (Textbox 1). The same reviewers will then independently review the full texts of potentially relevant articles. We will resolve any disagreements through discussion; if consensus cannot be reached, a third reviewer (JWS or SH) will make the final decision. We will record reasons for excluding studies at the full-text stage. We will document and report the entire process using a PRISMA flow diagram [29].

**Inclusion and exclusion criteria for study selection.**

**Inclusion criteria**

- Adults aged ≥60 years
- Frailty assessed using validated tools (Fried Frailty Phenotype, Frailty Index, Laboratory-based Frailty Index, Tilburg Frailty Indicator, Edmonton Frailty Scale)
- Biomarkers measured at baseline prior to frailty assessment
- Biomarkers from biological fluids (blood, urine, saliva, cerebrospinal fluid)
- Biomarker types: molecular, genetic, transcriptomic, functional imaging biomarkers
- Longitudinal study design with ≥2 time points
- Frailty incidence or progression assessed over time
- Peer-reviewed, full-text, published in English/Korean/Chinese

**Exclusion criteria**

- Animal studies or nonhuman data
- Frailty not defined or not measured as an outcome
- Studies assessing only frailty risk factors without frailty outcome
- Nonbiological measures (InBody, Bioelectrical Impedance Analysis)
- Imaging not related to physiological or neural function
- Longitudinal design but cross-sectional analysis only
- Preprints, abstracts, dissertations, or unavailable full-text

### Data Extraction

Two independent reviewers will extract data using a standardized form developed for this review. Extracted information will include study characteristics (author, year of publication, country, and study design), participant details (age and frailty assessment tools used), types of biomarkers measured at baseline, specimen used for measurement, follow-up duration, statistical or analytical methods, and key findings on the longitudinal association between biomarkers and frailty. When annual (or other interval) assessments are available, each follow-up period will be explicitly recorded to capture how biomarker trajectories and frailty status change over time, enabling clearer interpretation of the temporal relationships reported across studies.

We will refine the data extraction form as needed during the extraction process to ensure comprehensive capture of information relevant to the review questions. Discrepancies between the two reviewers (KS and YY) will be resolved through discussion. If consensus cannot be reached, a third reviewer (JWS) will make the final decision. For unclear or missing data, we will record “not reported” in the data extraction sheet. We will not apply imputation or substitution. These gaps will be acknowledged in the narrative synthesis.

### Data Synthesis

We will provide a narrative synthesis to describe how the findings align with the objectives and research questions of the review, following [30]. In the results section, we will synthesize the identified biomarkers and their longitudinal associations with frailty. We will stratify results by study population, including community-dwelling older adults and patients with metabolic disorders, musculoskeletal conditions, neurological diseases, cancer, infectious disease, and cardiovascular disease. Biomarkers will be categorized by specific lineage: inflammatory (eg, C-reactive protein, interleukin-6, tumor necrosis factor-α), metabolic (eg, glucose, hemoglobin A_1c_, lipid profiles), hormonal (eg, testosterone, sex hormone–binding globulin, insulin-like growth factor I), and other groups. We will create a summary figure to present the initial and follow-up periods for each study, enabling comparison of the longitudinal effects over time.

### Critical Appraisal

We will conduct this review using the JBI critical appraisal tools for cohort studies [31]. In this review, we will consider biomarkers as the exposure and frailty as the outcome. We will present the results of the critical appraisal in a summary table, which will show the total number and percentage of “yes” responses for each study. As this study is a scoping review, all relevant studies will be included regardless of methodological quality. However, we will provide a summary of the number of high-quality studies in the current evidence base.

### Ethical Considerations

Because this study relied exclusively on publicly available published and gray literature, ethics committee approval was not required.

## Results

**Figure 1:**
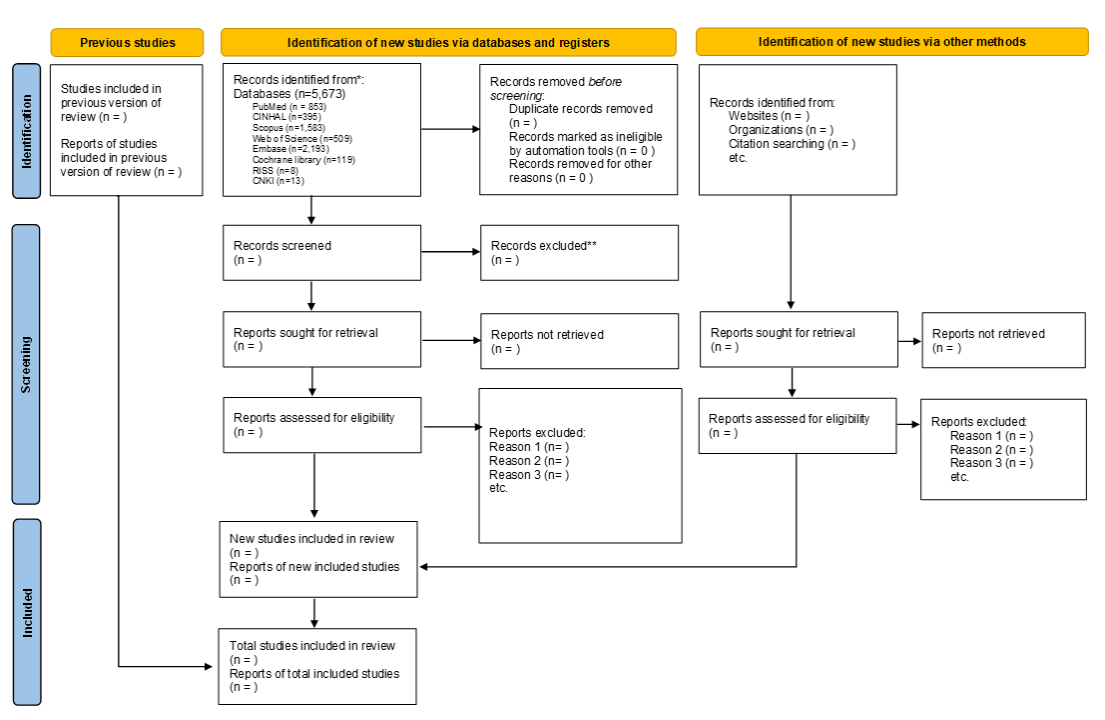
Flow diagram of identification, screening, and inclusion of studies for the scoping review.

We initiated an exploratory search in July 2024 to obtain preliminary results. After removing duplicates, the preliminary search identified 3315 records. We plan to complete this review by December 2026 (Figure 1).

## Discussion

### Anticipated Findings

Through this scoping review, we expect to identify and synthesize biomarkers of frailty that reflect the sequential influence of biological factors on frailty progression in older adults. We hypothesize that multiple heterogeneous biomarkers, including inflammatory, metabolic, cardiovascular, endocrine, and neurologic markers, exist. However, we also expect substantial variability in biomarker types, frailty definitions, follow-up durations, and statistical and analytic approaches across studies.

### Comparison to Prior Work

Previous reviews suggested albumin, hemoglobin, interlukin-6, and tumor necrosis factor-α as potential biomarkers for frailty and sarcopenia and reported inflammatory markers such as leukocyte, lymphocytes, C-reactive protein, interleukin-6, interleukin-10, and tumor necrosis factor-α as a biomarker for frailty in a cross-sectional study [10,32]; however, few longitudinal studies have been conducted, leaving a knowledge gap about the potential use of biomarkers of frailty for early detection and prevention. This review will address the range of biomarkers of frailty that precede frailty incidence or progression and overview existing findings by biological process and biomarker source, as well as follow-up duration and analytical and statistical methods.

### Strengths and Limitations

This review addresses the challenge of systematically examining the complex biological changes that precede frailty and evaluating their potential clinical relevance. By integrating current evidence without restricting the scope to predefined biomarker categories, this work captures the multifaceted biological processes underlying frailty and reflects its dynamic and systemic nature. Importantly, this approach allows for a more comprehensive understanding of frailty-related biological alterations across diverse physiological systems.

A key strength of this review lies in its inclusive exploration of heterogeneous biomarker types and analytical strategies. We examined a broad spectrum of biomarkers and analytical methodologies rather than focusing on a single class, thereby providing insight into how different approaches contribute to biomarker discovery and interpretation. This approach also enabled a critical discussion of methodological considerations, including study design, analytic frameworks, and appropriate follow-up durations needed to meaningfully capture longitudinal changes in frailty status.

Furthermore, by incorporating not only blood-based biomarkers but also neurologic markers such as fMRI, this review highlights the multidimensional nature of frailty. This broader biological perspective is particularly relevant for understanding cognitive frailty and other neurobiological dimensions of frailty that extend beyond traditional physical or inflammatory markers, thereby supporting a more holistic and personalized framework for frailty prediction and management.

Despite these strengths, several limitations should be acknowledged. As noted, most included studies were observational or cohort-based, limiting causal inference. In addition, substantial heterogeneity in biomarker assessment methods, statistical analyses, and outcome definitions precluded meta-analytic synthesis and the identification of statistically robust biomarkers. The lack of standardized protocols further limited direct comparisons across studies. Moreover, this review did not include body composition or functional biomarkers derived from measures such as bioelectrical impedance analysis (eg, InBody), which may restrict the scope of biological domains represented. Excluding such markers may underestimate the contribution of musculoskeletal and body composition-related pathways to frailty and sarcopenia, highlighting an important area for future integrative research.

### Future Directions

Future biomarker research should adopt a more integrative approach by combining molecular biomarkers with functional and body-composition measures such as bioelectrical impedance–derived indices, physical performance metrics, and wearable-based life-log data, while integrating multi-omics data (eg, genomics, transcriptomics, proteomics, and metabolomics) within a systems biology framework to better capture the complex biological mechanisms underlying frailty.

In addition, future studies should also identify and describe biomarker evidence specific to distinct frailty subgroups, including physical, cognitive, and affective frailty, to better reflect the heterogeneous biological pathways underlying frailty. For biomarkers that are consistently reported and show strong associations with frailty across studies, meta-analytic synthesis is needed to strengthen the evidence base and support clinical translation.

### Dissemination Plans

The findings of this scoping review will be disseminated through publication in a peer-reviewed journal, with manuscript submission anticipated in December 2026.

### Conclusions

This scoping review will systematically identify and examine longitudinal biomarkers that precede frailty in older adults, providing an integrated overview of the complex biological changes underlying frailty progression. By synthesizing evidence across diverse biomarker domains and analytical approaches, this review will emphasize the potential for advancing personalized frailty prediction and management while highlighting the multifaceted and dynamic nature of frailty biology.

## Appendix

Multimedia Appendix 1 Search trails.

## Abbreviations

fMRI: functional magnetic resonance imaging
JBI: Joanna Briggs Institute
MeSH: Medical Subject Headings
PRISMA-ScR: Preferred Reporting Items for Systematic Reviews and Meta-Analyses Extension for Scoping Reviews

## References

[1] Dlima SD, Hall A, Aminu AQ, Akpan A, Todd C, Vardy ERLC (2024). Frailty: a global health challenge in need of local action. BMJ Glob Health.

[2] Fried LP, Tangen CM, Walston J, Newman AB, Hirsch C, Gottdiener J, Seeman T, Tracy R, Kop WJ, Burke G, McBurnie MA, Cardiovascular Health Study Collaborative Research Group (2001). Frailty in older adults: evidence for a phenotype. J Gerontol A Biol Sci Med Sci.

[3] Gobbens RJ, van Assen MA, Luijkx KG, Schols JM (2012). Testing an integral conceptual model of frailty. J Adv Nurs.

[4] Sánchez-García Sergio, García-Peña Carmen, Salvà Antoni, Sánchez-Arenas Rosalinda, Granados-García Víctor, Cuadros-Moreno J, Velázquez-Olmedo Laura Bárbara, Cárdenas-Bahena Ángel (2017). Frailty in community-dwelling older adults: association with adverse outcomes. Clin Interv Aging.

[5] Chittrakul J, Siviroj P, Sungkarat S, Sapbamrer R (2020). Physical frailty and fall risk in community-dwelling older adults: a cross-sectional study. J Aging Res.

[6] Pearce J, Martin S, Heritage S, Khoury EG, Kucharczak J, Nuamek T, Cairns DA, Velikova G, Richards SH, Clegg A, Gilbert A (2025). Frailty and outcomes in adults undergoing systemic anticancer treatment: a systematic review and meta-analysis. J Natl Cancer Inst.

[7] Wleklik M, Denfeld Q, Czapla M, Jankowska EA, Piepoli MF, Uchmanowicz I (2023). A patient with heart failure, who is frail: how does this affect therapeutic decisions?. Cardiol J.

[8] Liu C, Yu J, Li X, Wei H, Liu X, Zhang W, Xu J (2025). Progress in lung cancer study coupled with cognitive frailty in elderly individuals. Geriatr Nurs.

[9] Faller JW, Pereira DDN, de Souza S, Nampo FK, Orlandi FDS, Matumoto S (2019). Instruments for the detection of frailty syndrome in older adults: A systematic review. PLoS One.

[10] Picca Anna, Coelho-Junior Hélio José, Calvani Riccardo, Marzetti Emanuele, Vetrano Davide Liborio (2022). Biomarkers shared by frailty and sarcopenia in older adults: A systematic review and meta-analysis. Ageing Res Rev.

[11] Clegg A, Young J, Iliffe S, Rikkert MO, Rockwood K (2013). Frailty in elderly people. Lancet.

[12] Avram Lucreția, Ungureanu Marius I, Crişan Dana, Donca Valer (2024). Assessment of frailty scores among geriatric patients hospitalized in the north-western region of Romania: a cross-sectional study. Medicina (Kaunas).

[13] Lin Kun-Pei, Li Hsin-Yi, Chen Jen-Hau, Lu Feng-Ping, Wen Chiung-Jung, Chou Yi-Chun, Wu Meng-Chen, Derrick Chan Ding-Cheng, Chen Yung-Ming (2023). Prediction of adverse health outcomes using an electronic frailty index among nonfrail and prefrail community elders. BMC Geriatr.

[14] Saedi AA, Feehan J, Phu S, Duque G (2019). Current and emerging biomarkers of frailty in the elderly. Clin Interv Aging.

[15] Wang Xiheng, Ji Jie (2025). Explainable machine learning framework for biomarker discovery by combining biological age and frailty prediction. Sci Rep.

[16] Yamasaki Takao, Tanaka Mutsuhide, Kumagai Shuzo (2025). Editorial: recent advances in research on cognitive frailty and related conditions. Front Aging Neurosci.

[17] Sepúlveda Magdalena, Arauna Diego, García Francisco, Albala Cecilia, Palomo Iván, Fuentes Eduardo (2022). Frailty in aging and the search for the optimal biomarker: a review. Biomedicines.

[18] Erusalimsky JD, Grillari J, Grune T, Jansen-Duerr P, Lippi G, Sinclair AJ, Tegnér Jesper, Viña Jose, Durrance-Bagale A, Miñambres Rebeca, Viegas M, Rodríguez-Mañas Leocadio, FRAILOMIC Consortium (2016). In search of 'omics'-based biomarkers to predict risk of frailty and its consequences in older individuals: the FRAILOMIC initiative. Gerontology.

[19] Kane AE, Sinclair DA (2019). Frailty biomarkers in humans and rodents: current approaches and future advances. Mech Ageing Dev.

[20] Cardoso AL, Fernandes A, Aguilar-Pimentel JA, de Angelis MH, Guedes JR, Brito MA, Ortolano S, Pani G, Athanasopoulou S, Gonos ES, Schosserer M, Grillari J, Peterson P, Tuna BG, Dogan S, Meyer A, van Os R, Trendelenburg A (2018). Towards frailty biomarkers: candidates from genes and pathways regulated in aging and age-related diseases. Ageing Res Rev.

[21] Gonçalves Rafaella Silva Dos Santos Aguiar, Maciel Álvaro Campos Cavalcanti, Rolland Yves, Vellas Bruno, de Souto Barreto Philipe (2022). Frailty biomarkers under the perspective of geroscience: a narrative review. Ageing Res Rev.

[22] Shi X, Yang H, Wang S, Yang Y, Li Y, Dou G, Ma Q (2025). Development and validation of a predictive nomogram for frailty based on thyroid function in older adults. Eur Geriatr Med.

[23] Rodriguez-Mañas L, Araujo de Carvalho I, Bhasin S, Bischoff-Ferrari HA, Cesari M, Evans W, Hare JM, Pahor M, Parini A, Rolland Y, Fielding RA, Walston J, Vellas B (2020). ICFSR task force perspective on biomarkers for sarcopenia and frailty. J Frailty Aging.

[24] Peters MDJ, Godfrey C, McInerney P, Munn Z, Tricco AC, Khalil H, Aromataris E, Lockwood C, Porritt K, Pilla B, Jordan Z (2024). JBI Manual for Evidence Synthesis.

[25] Tricco AC, Lillie E, Zarin W, O'Brien KK, Colquhoun H, Levac D, Moher D, Peters MD, Horsley T, Weeks L, Hempel S, Akl EA, Chang C, McGowan J, Stewart L, Hartling L, Aldcroft A, Wilson MG, Garritty C, Lewin S, Godfrey CM, Macdonald MT, Langlois EV, Soares-Weiser K, Moriarty J, Clifford T, Tunçalp Özge, Straus SE (2018). PRISMA Extension for Scoping Reviews (PRISMA-ScR): checklist and explanation. Ann Intern Med.

[26] Biomarkers. Sciences NIoEH.

[27] Leng S, Chen X, Mao G (2014). Frailty syndrome: an overview. CIA.

[28] Fried LP, Cohen AA, Xue Q, Walston J, Bandeen-Roche K, Varadhan R (2021). The physical frailty syndrome as a transition from homeostatic symphony to cacophony. Nat Aging.

[29] Page MJ, McKenzie JE, Bossuyt PM, Boutron I, Hoffmann TC, Mulrow CD, Shamseer L, Tetzlaff JM, Akl EA, Brennan SE, Chou R, Glanville J, Grimshaw JM, Hróbjartsson Asbjørn, Lalu MM, Li T, Loder EW, Mayo-Wilson E, McDonald S, McGuinness LA, Stewart LA, Thomas J, Tricco AC, Welch VA, Whiting P, Moher D (2021). The PRISMA 2020 statement: an updated guideline for reporting systematic reviews. BMJ.

[30] Campbell Mhairi, McKenzie Joanne E, Sowden Amanda, Katikireddi Srinivasa Vittal, Brennan Sue E, Ellis Simon, Hartmann-Boyce Jamie, Ryan Rebecca, Shepperd Sasha, Thomas James, Welch Vivian, Thomson Hilary (2020). Synthesis without meta-analysis (SWiM) in systematic reviews: reporting guideline. BMJ.

[31] Moola S, Munn Z, Tufanaru C, Aromataris E, Sears K, Sfetcu R, Currie M, Qureshi R, Mattis P, Lisy K, Mu P-F, Aromataris E, Munn Z (2020). Chapter 7: Systematic reviews of etiology and risk. JBI Manual for Evidence Synthesis.

[32] Xu Y, Wang M, Chen D, Jiang X, Xiong Z (2022). Inflammatory biomarkers in older adults with frailty: a systematic review and meta-analysis of cross-sectional studies. Aging Clin Exp Res.

