# Supplementary material for: Longitudinal Associations Between Biomarkers and Frailty in Older Adults: Protocol for a Scoping Review
Source: JMIR Res Protoc. 2026 Mar 24;15:e83312. doi: 10.2196/83312 (PMC13058529; doi:10.2196/83312)
Supplement: Multimedia Appendix 1 [file resprot_v15i1e83312_app1.docx]

Multimedia Appendix 1

Table S1. PubMed Search Trail

| Search # | Title OR Abstract OR MeSH Terms |  |
| --- | --- | --- |
| #1 | frail* OR Frailties[tiab] OR Frailness[tiab] OR Frailty Syndrome[tiab] OR Debility Debilities[tiab] OR prefrail[tiab] OR intermediate frail[tiab] OR frailty index[tiab] OR fried frailty phenotype[tiab] OR Clinical Frailty Scale[tiab] OR Edmonton Frail Scale[tiab] OR frailty[MH] |  |
| #2 | Longitudinal Studies[tiab] OR Longitudinal Study[tiab] OR Studies, Longitudinal[tiab] OR Study, Longitudinal[tiab] OR Longitudinal Survey[tiab] OR Longitudinal Surveys[tiab] OR Survey, Longitudinal[tiab] OR Surveys, Longitudinal[tiab] OR Prospective[tiab] OR Panel[tiab] OR Follow-up[tiab] OR Cohort[tiab] OR Longitudinal Studies[MH] |  |
| #3 | Marker, Biological[tiab] OR Biological Marker[tiab] OR Biologic Marker[tiab] OR Marker, Biologic[tiab] OR Biological Markers[tiab] OR Biologic Markers[tiab] OR Markers, Biologic[tiab] OR Biomarker[tiab] OR Markers, Biological[tiab] OR Markers, Immunologic[tiab] OR Immune Markers[tiab] OR Markers, Immune[tiab] OR Marker, Immunologic[tiab] OR Immunologic Markers[tiab] OR Immune Marker[tiab] OR Marker, Immune[tiab] OR Immunologic Marker[tiab] OR Serum Markers[tiab] OR Markers, Serum[tiab] OR Marker, Serum[tiab] OR Serum Marker[tiab] OR Markers, Clinical[tiab] OR Clinical Markers[tiab] OR Clinical Marker[tiab] OR Marker, Clinical[tiab] OR Biochemical Marker[tiab] OR Markers, Biochemical[tiab] OR Marker, Biochemical[tiab] OR Biochemical Markers[tiab] OR Markers, Laboratory[tiab] OR Laboratory Markers[tiab] OR Laboratory Marker[tiab] OR Marker, Laboratory[tiab] OR interleukin-6[tiab] OR tumor necrosis factor-alpha[tiab] OR C-Reactive Protein[tiab] OR myokines[tiab] OR insulin-like growth factor[tiab] OR Cyclase-associated protein 2[tiab] OR miRNA*[tiab] OR cytokine[tiab] OR inflammatory marker[tiab] OR proteomics[tiab] OR metabolite[tiab] OR interleukin 1[tiab] OR immunosenescence[tiab] OR inflammation[tiab] OR senescence markers[tiab] OR oxidative stress markers [tiab] OR chemokine* OR mechano-growth factor[tiab] OR Insulin growth factor-1[tiab] OR Homeostasis Model Assessment of Insulin Resistance[tiab] OR oxidized low-density lipoprotein[tiab] OR superoxide dismutase[tiab] OR glutathione peroxidase[tiab] OR Nuclear factor erythroid-2 related factor 2[tiab] OR Peroxisome proliferator-activated receptor-gamma coactivator 1-alpha[tiab] OR Sirtuin 1[tiab] OR Sirtuin 3 [tiab] OR Sirtuin 6 [tiab] OR soluble klotho[tiab] OR myostatin[tiab] OR follistatin[tiab] OR irisin[tiab] OR Dehydroepiandrosterone sulphate[tiab] OR Sex hormone-binding globulin[tiab] OR Biomarkers[MH] |  |
| #4 | English[lang] OR Korean [lang] OR Chinese[lang]）NOT (animals[Mesh:noexp] NOT (animals[Mesh:noexp] AND humans[Mesh])) NOT (Autobiography[ptyp] OR Bibliography[ptyp] OR Biography[ptyp] OR pubmed books[filter] OR Comment[ptyp] OR Dataset[ptyp] OR Dictionary[ptyp] OR Editorial[ptyp] OR Electronic Supplementary Materials[ptyp] OR Interview[ptyp] OR Legislation[ptyp] OR News[ptyp] OR Newspaper Article[ptyp] OR Retracted Publication[sb] OR Retraction of Publication[sb] OR Technical Report[ptyp] OR Letter[ptyp] OR Review[ptyp] |  |
| #5 | #1 AND #2 AND #3 AND #4 |  |

Table S2. CINAHL Search Trail

| Search # | Title OR Abstract OR MeSH Terms |  |
| --- | --- | --- |
| #1 | TI ( frail* OR Frailties OR Frailness OR Frailty Syndrome OR Debility Debilities OR prefrail OR intermediate frail OR frailty index OR fried frailty phenotype OR Clinical Frailty Scale OR Edmonton Frail Scale ) OR AB ( frail* OR Frailties OR Frailness OR Frailty Syndrome OR Debility Debilities OR prefrail OR intermediate frail OR frailty index OR fried frailty phenotype OR Clinical Frailty Scale OR Edmonton Frail Scale ) OR MH Frailty Syndrome |  |
| #2 | TI ( Longitudinal Studies OR Longitudinal Study OR Studies, Longitudinal OR Study, Longitudinal OR Longitudinal Survey OR Longitudinal Surveys OR Survey, Longitudinal OR Surveys, Longitudinal OR Prospective OR Panel OR Follow-up OR Cohort ) OR AB ( Longitudinal Studies OR Longitudinal Study OR Studies, Longitudinal OR Study, Longitudinal OR Longitudinal Survey OR Longitudinal Surveys OR Survey, Longitudinal OR Surveys, Longitudinal OR Prospective OR Panel OR Follow-up OR Cohort ) OR MH Prospective Studies |  |
| #3 | TI ( Marker, Biological OR Biological Marker OR Biologic Marker OR Marker, Biologic OR Biological Markers OR Biologic Markers OR Markers, Biologic OR Biomarker OR Markers, Biological OR Markers, Immunologic OR Immune Markers OR Markers, Immune OR Marker, Immunologic OR Immunologic Markers OR Immune Marker OR Marker, Immune OR Immunologic Marker OR Serum Markers OR Markers, Serum OR Marker, Serum OR Serum Marker OR Markers, Clinical OR Clinical Markers OR Clinical Marker OR Marker, Clinical OR Biochemical Marker OR Markers, Biochemical OR Marker, Biochemical OR Biochemical Markers OR Markers, Laboratory OR Laboratory Markers OR Laboratory Marker OR Marker, Laboratory OR interleukin-6 OR tumor necrosis factor-alpha OR C-Reactive Protein OR myokines OR insulin-like growth factor OR Cyclase-associated protein 2 OR miRNA* OR cytokine OR inflammatory marker OR proteomics OR metabolite OR interleukin 1 OR immunosenescence OR inflammation OR senescence markers OR oxidative stress markers OR chemokine* OR mechano-growth factor OR Insulin growth factor-1 OR Homeostasis Model Assessment of Insulin Resistance OR oxidized low-density lipoprotein OR superoxide dismutase OR glutathione peroxidase OR Nuclear factor erythroid-2 related factor 2 OR Peroxisome proliferator-activated receptor-gamma coactivator 1-alpha OR Sirtuin 1 OR Sirtuin 3 OR Sirtuin 6 OR soluble klotho OR myostatin OR follistatin OR irisin OR Dehydroepiandrosterone sulfate OR Sex hormone-binding globulin ) OR AB ( Marker, Biological OR Biological Marker OR Biologic Marker OR Marker, Biologic OR Biological Markers OR Biologic Markers OR Markers, Biologic OR Biomarker OR Markers, Biological OR Markers, Immunologic OR Immune Markers OR Markers, Immune OR Marker, Immunologic OR Immunologic Markers OR Immune Marker OR Marker, Immune OR Immunologic Marker OR Serum Markers OR Markers, Serum OR Marker, Serum OR Serum Marker OR Markers, Clinical OR Clinical Markers OR Clinical Marker OR Marker, Clinical OR Biochemical Marker OR Markers, Biochemical OR Marker, Biochemical OR Biochemical Markers OR Markers, Laboratory OR Laboratory Markers OR Laboratory Marker OR Marker, Laboratory OR interleukin-6 OR tumor necrosis factor-alpha OR C-Reactive Protein OR myokines OR insulin-like growth factor OR Cyclase-associated protein 2 OR miRNA* OR cytokine OR inflammatory marker OR proteomics OR metabolite OR interleukin 1 OR immunosenescence OR inflammation OR senescence markers OR oxidative stress markers OR chemokine* OR mechano-growth factor OR Insulin growth factor-1 OR Homeostasis Model Assessment of Insulin Resistance OR oxidized low-density lipoprotein OR superoxide dismutase OR glutathione peroxidase OR Nuclear factor erythroid-2 related factor 2 OR Peroxisome proliferator-activated receptor-gamma coactivator 1-alpha OR Sirtuin 1 OR Sirtuin 3 OR Sirtuin 6 OR soluble klotho OR myostatin OR follistatin OR irisin OR Dehydroepiandrosterone sulfate OR Sex hormone-binding globulin ) OR MH Biological Markers |  |
| #4 | TX (English OR Korean OR Chinese） NOT (animals NOT MH (animals AND humans)) NOT (Autobiography OR Bibliography OR Biography OR pubmed books OR Comment OR Dataset OR Dictionary OR Editorial OR Electronic Supplementary Materials OR Interview OR Legislation OR News OR Newspaper Article OR Retracted Publication OR Retraction of Publication OR Technical Report OR Letter OR Review) |  |
| #5 | #1 AND #2 AND #3 AND #4 |  |

Table S3. Scopus Search Trail

| Search # | Title OR Abstract OR Keywords |  |
| --- | --- | --- |
| #1 | frail* OR Frailties OR Frailness OR "Frailty Syndrome" OR Debility OR Debilities OR prefrail OR "intermediate frail" OR "frailty index" OR "fried frailty phenotype" OR "Clinical Frailty Scale" OR "Edmonton Frail Scale" |  |
| #2 | “Longitudinal Studies” OR “Longitudinal Study” OR “Studies, Longitudinal” OR “Study, Longitudinal” OR “Longitudinal Survey” OR “Longitudinal Surveys” OR “Survey, Longitudinal” OR “Surveys, Longitudinal” OR “Prospective” OR “Panel” OR “Follow-up” OR Cohort |  |
| #3 | "Marker, Biological" OR "Biological Marker" OR "Biologic Marker" OR "Marker, Biologic" OR "Biological Markers" OR "Biologic Markers" OR "Markers, Biologic" OR "Biomarker" OR "Markers, Biological" OR "Markers, Immunologic" OR "Immune Markers" OR "Markers, Immune" OR "Marker, Immunologic" OR "Immunologic Markers" OR "Immune Marker" OR "Marker, Immune" OR "Immunologic Marker" OR "Serum Markers" OR "Markers, Serum" OR "Marker, Serum" OR "Serum Marker" OR "Markers, Clinical" OR "Clinical Markers" OR "Clinical Marker" OR "Marker, Clinical" OR "Biochemical Marker" OR "Markers, Biochemical" OR "Marker, Biochemical" OR "Biochemical Markers" OR "Markers, Laboratory" OR "Laboratory Markers" OR "Laboratory Marker" OR "Marker, Laboratory" OR "interleukin-6" OR "tumor necrosis factor-alpha" OR "C-Reactive Protein" OR "myokines" OR "insulin-like growth factor" OR "Cyclase-associated protein 2" OR miRNA* OR "cytokine" OR "inflammatory marker" OR "proteomics" OR "metabolite" OR "interleukin 1" OR "immunosenescence" OR "inflammation" OR "senescence markers" OR "oxidative stress markers" OR chemokine* OR "mechano-growth factor" OR "Insulin growth factor-1" OR "Homeostasis Model Assessment of Insulin Resistance" OR "oxidized low-density lipoprotein" OR "superoxide dismutase" OR "glutathione peroxidase" OR "Nuclear factor erythroid-2 related factor 2" OR "Peroxisome proliferator-activated receptor-gamma coactivator 1-alpha" OR "Sirtuin 1" OR "Sirtuin 3" OR "Sirtuin 6" OR "soluble klotho" OR "myostatin" OR "follistatin" OR "irisin" OR "Dehydroepiandrosterone sulfate" OR "Sex hormone-binding globulin" |  |
| #4 | ( LANGUAGE ( english OR chinese OR korean ) AND NOT TITLE-ABS-KEY ( animals )) AND ( LIMIT-TO ( DOCTYPE , "ar" ) ) |  |
| #5 | #1 AND #2 AND #3 AND #4 |  |
|  |  |  |

Table S4. Web of science Search Trail

| Search # | Searches title OR Abstract OR Keyword plus | Articles Revealed |
| --- | --- | --- |
| #1 | (TI=(frail* OR Frailties OR Frailness OR "Frailty Syndrome" OR Debility OR Debilities OR prefrail OR "intermediate frail" OR "frailty index" OR "fried frailty phenotype" OR "Clinical Frailty Scale" OR "Edmonton Frail Scale")) OR AB=(frail* OR Frailties OR Frailness OR "Frailty Syndrome" OR Debility OR Debilities OR prefrail OR "intermediate frail" OR "frailty index" OR "fried frailty phenotype" OR "Clinical Frailty Scale" OR "Edmonton Frail Scale") |  |
| #2 | (TI=( “Longitudinal Studies” OR “Longitudinal Study” OR “Studies, Longitudinal” OR “Study, Longitudinal” OR “Longitudinal Survey” OR “Longitudinal Surveys” OR “Survey, Longitudinal” OR “Surveys, Longitudinal” OR “Prospective” OR “Panel” OR “Follow-up” OR Cohort)) OR AB=( “Longitudinal Studies” OR “Longitudinal Study” OR “Studies, Longitudinal” OR “Study, Longitudinal” OR “Longitudinal Survey” OR “Longitudinal Surveys” OR “Survey, Longitudinal” OR “Surveys, Longitudinal” OR “Prospective” OR “Panel” OR “Follow-up” OR Cohort) |  |
| #3 | (TI=("Marker, Biological" OR "Biological Marker" OR "Biologic Marker" OR "Marker, Biologic" OR "Biological Markers" OR "Biologic Markers" OR "Markers, Biologic" OR "Biomarker" OR "Markers, Biological" OR "Markers, Immunologic" OR "Immune Markers" OR "Markers, Immune" OR "Marker, Immunologic" OR "Immunologic Markers" OR "Immune Marker" OR "Marker, Immune" OR "Immunologic Marker" OR "Serum Markers" OR "Markers, Serum" OR "Marker, Serum" OR "Serum Marker" OR "Markers, Clinical" OR "Clinical Markers" OR "Clinical Marker" OR "Marker, Clinical" OR "Biochemical Marker" OR "Markers, Biochemical" OR "Marker, Biochemical" OR "Biochemical Markers" OR "Markers, Laboratory" OR "Laboratory Markers" OR "Laboratory Marker" OR "Marker, Laboratory" OR "interleukin-6" OR "tumor necrosis factor-alpha" OR "C-Reactive Protein" OR "myokines" OR "insulin-like growth factor" OR "Cyclase-associated protein 2" OR miRNA* OR "cytokine" OR "inflammatory marker" OR "proteomics" OR "metabolite" OR "interleukin 1" OR "immunosenescence" OR "inflammation" OR "senescence markers" OR "oxidative stress markers" OR chemokine* OR "mechano-growth factor" OR "Insulin growth factor-1" OR "Homeostasis Model Assessment of Insulin Resistance" OR "oxidized low-density lipoprotein" OR "superoxide dismutase" OR "glutathione peroxidase" OR "Nuclear factor erythroid-2 related factor 2" OR "Peroxisome proliferator-activated receptor-gamma coactivator 1-alpha" OR "Sirtuin 1" OR "Sirtuin 3" OR "Sirtuin 6" OR "soluble klotho" OR "myostatin" OR "follistatin" OR "irisin" OR "Dehydroepiandrosterone sulfate" OR "Sex hormone-binding globulin")) OR AB=("Marker, Biological" OR "Biological Marker" OR "Biologic Marker" OR "Marker, Biologic" OR "Biological Markers" OR "Biologic Markers" OR "Markers, Biologic" OR "Biomarker" OR "Markers, Biological" OR "Markers, Immunologic" OR "Immune Markers" OR "Markers, Immune" OR "Marker, Immunologic" OR "Immunologic Markers" OR "Immune Marker" OR "Marker, Immune" OR "Immunologic Marker" OR "Serum Markers" OR "Markers, Serum" OR "Marker, Serum" OR "Serum Marker" OR "Markers, Clinical" OR "Clinical Markers" OR "Clinical Marker" OR "Marker, Clinical" OR "Biochemical Marker" OR "Markers, Biochemical" OR "Marker, Biochemical" OR "Biochemical Markers" OR "Markers, Laboratory" OR "Laboratory Markers" OR "Laboratory Marker" OR "Marker, Laboratory" OR "interleukin-6" OR "tumor necrosis factor-alpha" OR "C-Reactive Protein" OR "myokines" OR "insulin-like growth factor" OR "Cyclase-associated protein 2" OR miRNA* OR "cytokine" OR "inflammatory marker" OR "proteomics" OR "metabolite" OR "interleukin 1" OR "immunosenescence" OR "inflammation" OR "senescence markers" OR "oxidative stress markers" OR chemokine* OR "mechano-growth factor" OR "Insulin growth factor-1" OR "Homeostasis Model Assessment of Insulin Resistance" OR "oxidized low-density lipoprotein" OR "superoxide dismutase" OR "glutathione peroxidase" OR "Nuclear factor erythroid-2 related factor 2" OR "Peroxisome proliferator-activated receptor-gamma coactivator 1-alpha" OR "Sirtuin 1" OR "Sirtuin 3" OR "Sirtuin 6" OR "soluble klotho" OR "myostatin" OR "follistatin" OR "irisin" OR "Dehydroepiandrosterone sulfate" OR "Sex hormone-binding globulin") |  |
| #4 | (((LA=(English OR Chinese OR Korean)) NOT TS=(animals)) NOT TS=(Autobiography OR Bibliography OR Biography OR “pubmed books” OR Comment OR Dataset OR Dictionary OR Editorial OR “Electronic Supplementary Materials” OR Interview OR Legislation OR News OR “Newspaper Article” OR “Retracted Publication” OR “Retraction of Publication” OR “Technical Report” OR Letter OR Review)) AND TS=(humans) |  |
| #5 | #1 AND #2 AND #3 AND #4 |  |

Table S5. Embase Search Trail

| Search # | Title OR Abstract OR Keywords |  |
| --- | --- | --- |
| #1 | frail*:ab,ti OR frailties:ab,ti OR frailness:ab,ti OR 'frailty syndrome':ab,ti OR debility:ab,ti OR debilities:ab,ti OR prefrail:ab,ti OR 'intermediate frail':ab,ti OR 'frailty index':ab,ti OR 'fried frailty phenotype':ab,ti OR 'clinical frailty scale':ab,ti OR 'edmonton frail scale':ab,ti OR 'frailty'/exp |  |
| #2 | 'longitudinal studies':ab,ti OR 'longitudinal study':ab,ti OR 'studies, longitudinal':ab,ti OR 'study, longitudinal':ab,ti OR 'longitudinal survey':ab,ti OR 'longitudinal surveys':ab,ti OR 'survey, longitudinal':ab,ti OR 'surveys, longitudinal':ab,ti OR 'prospective':ab,ti OR 'panel':ab,ti OR 'follow-up':ab,ti OR cohort:ab,ti OR 'longitudinal study'/exp |  |
| #3 | 'marker, biological':ab,ti OR 'biological marker':ab,ti OR 'biologic marker':ab,ti OR 'marker, biologic':ab,ti OR 'biological markers':ab,ti OR 'biologic markers':ab,ti OR 'markers, biologic':ab,ti OR 'biomarker':ab,ti OR 'markers, biological':ab,ti OR 'markers, immunologic':ab,ti OR 'immune markers':ab,ti OR 'markers, immune':ab,ti OR 'marker, immunologic':ab,ti OR 'immunologic markers':ab,ti OR 'immune marker':ab,ti OR 'marker, immune':ab,ti OR 'immunologic marker':ab,ti OR 'serum markers':ab,ti OR 'markers, serum':ab,ti OR 'marker, serum':ab,ti OR 'serum marker':ab,ti OR 'markers, clinical':ab,ti OR 'clinical markers':ab,ti OR 'clinical marker':ab,ti OR 'marker, clinical':ab,ti OR 'biochemical marker':ab,ti OR 'markers, biochemical':ab,ti OR 'marker, biochemical':ab,ti OR 'biochemical markers':ab,ti OR 'markers, laboratory':ab,ti OR 'laboratory markers':ab,ti OR 'laboratory marker':ab,ti OR 'marker, laboratory':ab,ti OR 'interleukin-6':ab,ti OR 'tumor necrosis factor-alpha':ab,ti OR 'c-reactive protein':ab,ti OR 'myokines':ab,ti OR 'insulin-like growth factor':ab,ti OR 'cyclase-associated protein 2':ab,ti OR mirna*:ab,ti OR 'cytokine':ab,ti OR 'inflammatory marker':ab,ti OR 'proteomics':ab,ti OR 'metabolite':ab,ti OR 'interleukin 1':ab,ti OR 'immunosenescence':ab,ti OR 'inflammation':ab,ti OR 'senescence markers':ab,ti OR 'oxidative stress markers':ab,ti OR chemokine*:ab,ti OR 'mechano-growth factor':ab,ti OR 'insulin growth factor-1':ab,ti OR 'homeostasis model assessment of insulin resistance':ab,ti OR 'oxidized low-density lipoprotein':ab,ti OR 'superoxide dismutase':ab,ti OR 'glutathione peroxidase':ab,ti OR 'nuclear factor erythroid-2 related factor 2':ab,ti OR 'peroxisome proliferator-activated receptor-gamma coactivator 1-alpha':ab,ti OR 'sirtuin 1':ab,ti OR 'sirtuin 3':ab,ti OR 'sirtuin 6':ab,ti OR 'soluble klotho':ab,ti OR 'myostatin':ab,ti OR 'follistatin':ab,ti OR 'irisin':ab,ti OR 'dehydroepiandrosterone sulfate':ab,ti OR 'sex hormone-binding globulin':ab,ti OR 'marker'/exp |  |
| #4 | (english:la OR korean:la OR chinese:la) NOT (autobiography:it OR bibliography:it OR biography:it OR 'pubmed books':it OR comment:it OR dataset:it OR dictionary:it OR editorial:it OR 'electronic supplementary materials':it OR interview:it OR legislation:it OR news:it OR 'newspaper article':it OR 'retracted publication':it OR 'retraction of publication':it OR 'technical report':it OR letter:it OR review:it) NOT animals:ti,ab,kw |  |
| #5 | #1 AND #2 AND #3 AND #4 |  |

Table S6. Cochrane library Search Trail (Search updated 07/Aug/2024)

| Search # | Title OR Abstract OR MeSH Terms |  |
| --- | --- | --- |
| #1 | frail* OR Frailties OR Frailness OR "Frailty Syndrome" OR Debility OR Debilities OR prefrail OR "intermediate frail" OR "frailty index" OR "fried frailty phenotype" OR "Clinical Frailty Scale" OR "Edmonton Frail Scale" OR MH(Frailty Syndrome) |  |
| #2 | “Longitudinal Studies” OR “Longitudinal Study” OR “Studies, Longitudinal” OR “Study, Longitudinal” OR “Longitudinal Survey” OR “Longitudinal Surveys” OR “Survey, Longitudinal” OR “Surveys, Longitudinal” OR “Prospective” OR “Panel” OR “Follow-up” OR Cohort OR MH(Prospective Studies) |  |
| #3 | "Marker, Biological" OR "Biological Marker" OR "Biologic Marker" OR "Marker, Biologic" OR "Biological Markers" OR "Biologic Markers" OR "Markers, Biologic" OR "Biomarker" OR "Markers, Biological" OR "Markers, Immunologic" OR "Immune Markers" OR "Markers, Immune" OR "Marker, Immunologic" OR "Immunologic Markers" OR "Immune Marker" OR "Marker, Immune" OR "Immunologic Marker" OR "Serum Markers" OR "Markers, Serum" OR "Marker, Serum" OR "Serum Marker" OR "Markers, Clinical" OR "Clinical Markers" OR "Clinical Marker" OR "Marker, Clinical" OR "Biochemical Marker" OR "Markers, Biochemical" OR "Marker, Biochemical" OR "Biochemical Markers" OR "Markers, Laboratory" OR "Laboratory Markers" OR "Laboratory Marker" OR "Marker, Laboratory" OR "interleukin-6" OR "tumor necrosis factor-alpha" OR "C-Reactive Protein" OR "myokines" OR "insulin-like growth factor" OR "Cyclase-associated protein 2" OR miRNA* OR "cytokine" OR "inflammatory marker" OR "proteomics" OR "metabolite" OR "interleukin 1" OR "immunosenescence" OR "inflammation" OR "senescence markers" OR "oxidative stress markers" OR chemokine* OR "mechano-growth factor" OR "Insulin growth factor-1" OR "Homeostasis Model Assessment of Insulin Resistance" OR "oxidized low-density lipoprotein" OR "superoxide dismutase" OR "glutathione peroxidase" OR "Nuclear factor erythroid-2 related factor 2" OR "Peroxisome proliferator-activated receptor-gamma coactivator 1-alpha" OR "Sirtuin 1" OR "Sirtuin 3" OR "Sirtuin 6" OR "soluble klotho" OR "myostatin" OR "follistatin" OR "irisin" OR "Dehydroepiandrosterone sulfate" OR "Sex hormone-binding globulin" OR MH(Biological Markers) |  |
| #4 | English OR Korean OR Chinese NOT (animals OR (MH animals AND MH humans)) NOT (Autobiography OR Bibliography OR Biography OR "pubmed books" OR Comment OR Dataset OR Dictionary OR Editorial OR "Electronic Supplementary Materials" OR Interview OR Legislation OR News OR "Newspaper Article" OR Retracted Publication OR Retraction of Publication OR Technical Report OR Letter OR Review) |  |
| #5 | #1 AND #2 AND #3 AND #4 |  |

Table S7. RISS Search Trail

| Search # | All (전체) |  |
| --- | --- | --- |
| #1 | ( 전체 : Frail <OR> 전체 : Frailties <OR> 전체 : Frailness <OR> 전체 : Frailty Syndrome <OR> 전체 : Debility <OR> 전체 : Debilities <OR> 전체 : 노쇠 <OR> 전체 : 허약 ) ( 전체 : Longitudinal <OR> 전체 : Prospective <OR> 전체 : Panel <OR> 전체 : Follow-up <OR> 전체 : Cohort <OR> 전체 : 종단 <OR> 전체 : 종단적 <OR> 전체 : 코호트 <OR> 전체 : 패널 <OR> 전체 : 전향적 ) ( 전체 : Biological Marker <OR> 전체 : Biologic Marker <OR> 전체 : Biomarker <OR> 전체 : Immunologic Markers <OR> 전체 : Immune Marker <OR> 전체 : Serum Markers <OR> 전체 : Clinical Markers <OR> 전체 : 염증지표 <OR> 전체 : 생체지표 <OR> 전체 : 바이오마커) |  |

Table S8. CNKI（中国知网） Search Trail

| Search # | Title, Keyword, Title, Keyword and Abstract |  |
| --- | --- | --- |
| #1 | ((TI = ''Frail' + 'Frailties' + 'Frailness' + 'Frailty Syndrome' + 'Debility' + 'Debilities' + 'prefrail' +'intermediate frail' + 'frailty index' + 'fried frailty phenotype' + 'Clinical Frailty Scale' + 'Edmonton Frail Scale'+ '衰弱' + '虚弱' OR AB = ''Frail' + 'Frailties' + 'Frailness' + 'Frailty Syndrome'+ 'Debility' + 'Debilities' + 'prefrail' + 'intermediate frail' + 'frailty index' + 'fried frailty phenotype' + 'Clinical Frailty Scale' + 'Edmonton Frail Scale'+ '衰弱' + '虚弱'' )) AND ((TI = ' 'Longitudinal Studies' + 'Longitudinal Study' + 'Studies, Longitudinal'+ 'Study, Longitudinal' + 'Longitudinal Survey' + 'Longitudinal Surveys'+ 'Survey, Longitudinal' + 'Surveys, Longitudinal' + 'Prospective' + 'Panel' + 'Follow-up' + 'Cohort' + '纵向' +'前瞻性'+'队列'') OR (AB = ' 'Longitudinal Studies' + 'Longitudinal Study' + 'Studies, Longitudinal'+ 'Study, Longitudinal' + 'Longitudinal Survey' + 'Longitudinal Surveys'+ 'Survey, Longitudinal' + 'Surveys, Longitudinal' + 'Prospective' + 'Panel' + 'Follow-up' + 'Cohort' + '纵向' +'前瞻性'+'队列'')) AND ((TI = '''Marker, Biological' + 'Biological Marker' + 'Biologic Marker' + 'Marker, Biologic' + 'Biological Markers' + 'Biologic Markers' + 'Markers, Biologic' + 'Biomarker' + 'Markers, Biological' + 'Markers, Immunologic' + 'Immune Markers' + 'Markers, Immune' + 'Marker, Immunologic' + 'Immunologic Markers' + 'Immune Marker' + 'Marker, Immune' + 'Immunologic Marker' + 'Serum Markers' + 'Markers, Serum' + 'Marker, Serum' + 'Serum Marker' + 'Markers, Clinical' + 'Clinical Markers' + 'Clinical Marker' + 'Marker, Clinical' + 'Biochemical Marker' + 'Markers, Biochemical' + 'Marker, Biochemical' + 'Biochemical Markers' + 'Markers, Laboratory' + 'Laboratory Markers' + 'Laboratory Marker' + 'Marker, Laboratory' + 'interleukin-6' + 'tumor necrosis factor-alpha' + 'C-Reactive Protein' + 'myokines' + 'insulin-like growth factor' + 'Cyclase-associated protein 2' + 'miRNA' + 'cytokine' + 'inflammatory marker' + 'proteomics' + 'metabolite' + 'interleukin 1' + 'immunosenescence' + 'inflammation' + 'senescence markers' + 'oxidative stress markers' + 'chemokine' + 'mechano-growth factor' + 'Insulin growth factor-1' + 'Homeostasis Model Assessment of Insulin Resistance' + 'oxidized low-density lipoprotein' + 'superoxide dismutase' + 'glutathione peroxidase' + 'Nuclear factor erythroid-2 related factor 2' + 'Peroxisome proliferator-activated receptor-gamma coactivator 1-alpha' + 'Sirtuin 1' + 'Sirtuin 3' + 'Sirtuin 6' + 'soluble klotho' + 'myostatin' + 'follistatin' + 'irisin' + 'Dehydroepiandrosterone sulphate' + 'Sex hormone-binding globulin'+ '生物指标' + '生物标志' + ‘免疫指标' + '血清标志物' + 'C反应蛋白' + '炎症' + '临床标记' + '细胞因子' + '氧化应激'') OR (AB = '''Marker, Biological' + 'Biological Marker' + 'Biologic Marker' + 'Marker, Biologic'  + 'Biological Markers' + 'Biologic Markers' + 'Markers, Biologic' + 'Biomarker' + 'Markers, Biological' + 'Markers, Immunologic' + 'Immune Markers' + 'Markers, Immune' + 'Marker, Immunologic' + 'Immunologic Markers' + 'Immune Marker' + 'Marker, Immune' + 'Immunologic Marker' + 'Serum Markers' + 'Markers, Serum' + 'Marker, Serum' + 'Serum Marker' + 'Markers, Clinical' + 'Clinical Markers' + 'Clinical Marker' + 'Marker, Clinical' + 'Biochemical Marker' + 'Markers, Biochemical' + 'Marker, Biochemical' + 'Biochemical Markers' + 'Markers, Laboratory' + 'Laboratory Markers' + 'Laboratory Marker' + 'Marker, Laboratory' + 'interleukin-6' + 'tumor necrosis factor-alpha' + 'C-Reactive Protein' + 'myokines' + 'insulin-like growth factor' + 'Cyclase-associated protein 2' + 'miRNA' + 'cytokine' + 'inflammatory marker' + 'proteomics' + 'metabolite' + 'interleukin 1' + 'immunosenescence' + 'inflammation' + 'senescence markers' + 'oxidative stress markers' + 'chemokine' + 'mechano-growth factor' + 'Insulin growth factor-1' + 'Homeostasis Model Assessment of Insulin Resistance' + 'oxidized low-density lipoprotein' + 'superoxide dismutase' + 'glutathione peroxidase' + 'Nuclear factor erythroid-2 related factor 2' + 'Peroxisome proliferator-activated receptor-gamma coactivator 1-alpha' + 'Sirtuin 1' + 'Sirtuin 3' + 'Sirtuin 6' + 'soluble klotho' + 'myostatin' + 'follistatin' + 'irisin' + 'Dehydroepiandrosterone sulphate' + 'Sex hormone-binding globulin'+ '生物指标' + '生物标志' + ‘免疫指标' + '血清标志物' + 'C反应蛋白' + '炎症' + '临床标记' + '细胞因子' + '氧化应激')) AND ( TI % 'animal experiment' + 'rat' + 'mouse' + 'mice' + 'rabbit' + 'canine' + 'cat' OR AB % 'animal experiment' + 'rat' + 'mouse' + 'mice' + 'rabbit' + 'canine' + 'cat') AND NOT ( TI % 'human' OR AB % 'human' )) AND NOT (TI= 'animal experiment' + 'rat' + 'mouse' + 'mice' + 'rabbit' + 'canine' + 'cat'OR AB % 'animal experiment' + 'rat' + 'mouse' + 'mice' + 'rabbit' + 'canine' + 'cat') AND NOT (TI = 'human' OR AB = 'human' ) AND NOT (PT = 'autobiography' + 'bibliography' + 'biography' + 'comment' + 'dataset'+ 'dictionary' + 'editorial' + 'interview' + 'legislation' + 'news'+ 'newspaper article' + 'retracted publication' + 'retraction' + 'technical report' + 'letter' + 'review' + 'book' ) |  |
